# Functional insights into a putative 2-OG oxygenase involved in jasmonic acid metabolism and xylem development in *Populus trichocarpa*

**DOI:** 10.1093/pcp/pcag035

**Published:** 2026-03-16

**Authors:** Natalia Wojciechowska, Maciej Janicki, Katarzyna Marzec-Schmidt, Thierry Heitz, Magdalena Ślachetka, Agnieszka Ludwików, Agnieszka Bagniewska-Zadworna

**Affiliations:** Department of General Botany, Faculty of Biology, Institute of Experimental Biology, Adam Mickiewicz University, Uniwersytetu Poznańskiego 6, 61-614 Poznań, Poland; Laboratory of Biotechnology, Faculty of Biology, Institute of Molecular Biology and Biotechnology, Adam Mickiewicz University, Uniwersytetu Poznańskiego 6, 61-614 Poznań, Poland; Findus Sverige AB, Nomad Foods Ltd, Sellebergavägen 13, 267 40 Bjuv, Skåne County, Sweden; Centre National de la Recherche Scientifique (IBMP CNRS), Institut de Biologie Moléculaire des Plantes, Université de Strasbourg, 12 rue du Général Zimmer, 67000 Strasbourg, France; Department of General Botany, Faculty of Biology, Institute of Experimental Biology, Adam Mickiewicz University, Uniwersytetu Poznańskiego 6, 61-614 Poznań, Poland; Laboratory of Biotechnology, Faculty of Biology, Institute of Molecular Biology and Biotechnology, Adam Mickiewicz University, Uniwersytetu Poznańskiego 6, 61-614 Poznań, Poland; Department of General Botany, Faculty of Biology, Institute of Experimental Biology, Adam Mickiewicz University, Uniwersytetu Poznańskiego 6, 61-614 Poznań, Poland

**Keywords:** 2-oxoglutarate (2-OG) and Fe(II)-dependent oxygenases, jasmonic acid oxidases, jasmonate-induced oxygenases, jasmonic acid, secondary growth, wood, xylogenesis

## Abstract

The xylem vascular tissue constitutes an efficient system of long-distance transport of water and nutrients, as well as structural support to the plant body. Despite its importance, the molecular mechanisms regulating xylogenesis remain only partially understood. In this study, we investigated two genes from *Populus trichocarpa* encoding 2-oxoglutarate (2-OG) and Fe(II)-dependent oxygenases, whose expression is significantly upregulated during secondary growth. Proteins encoded by those genes share similarities with *Arabidopsis* enzymes known as jasmonic acid oxygenases (JAOs) or jasmonate-induced oxygenases, which are involved in the hydroxylation of jasmonic acid (JA)-a hormone precursor critical for plant development and stress responses. We hypothesized that the *Populus* 2-OG oxygenases participate in JA metabolism and thereby influence wood formation. Their *in silico* structural analysis indicated that one of these proteins likely binds JA with similar efficiency to known JAO proteins. A link was supported by *in vitro* and *in vivo* experiments. Studies in an *Arabidopsis* JAO-depleted mutant revealed altered stem anatomy, characterized by a reduced stem diameter and an increased proportion of xylem area relative to the total stem cross-section, suggesting that disrupted JA metabolism can interfere with normal vascular development. These findings provide new insights into the hormonal regulation of vascular development and may have practical implications for improving wood quality or stress resilience in trees.

## Introduction

Plants undergo both primary and secondary growth to adapt to their environment and ensure survival. While primary growth increases the length of roots and shoots, secondary growth is responsible for increasing plant girth, particularly in woody species. Secondary growth results from the activity of two lateral meristems: the phellogen (cork cambium) and the vascular cambium (Inácio et al. 2022, Turley and Etchells 2022). The former produces the phellem (cork) outward and the phelloderm inward, together forming the three-layered periderm, which serves as the plant’s first line of defense against environmental stresses (Campilho et al. 2020, Serra et al. 2022). The vascular cambium divides periclinally, generating secondary xylem (also referred to as “wood”) internally and secondary phloem externally (Carlsbecker and Augstein 2021, Inácio et al. 2022).

The process of wood formation, referred to as “xylogenesis”, consists of a highly coordinated series of events that can be divided into several stages: cambial cell division, cell elongation, cell wall thickening, lignification, and programmed cell death (PCD) (Fukuda 1996, Turner et al. 2007). Mature secondary xylem is a structurally complex and heterogeneous tissue composed of various cell types with specialized shapes and functions, including tracheary elements (TEs), fibers, and parenchyma cells. TEs, which are dead and hollow at maturity, represent a critical wood cell type that, when axially connected, forms a continuous pathway for water and mineral transport. These cells withstand high hydrostatic pressure due to their thick, lignified secondary cell walls (SCWs) (Pittermann 2010, Schuetz et al. 2013, Słupianek et al. 2021).

The mechanisms and regulation of the specific stages of xylogenesis have long been the focus of scientific investigation. Early research primarily addressed the cytological and biochemical changes associated with this process (Esau et al. 1963, Cronshaw and Bouck 1965, Wodzicki and Humphreys 1973, Ye and Varner 1996). Transmission electron microscopy enabled detailed analysis of ultrastructural changes in differentiating wood cells, including SCW development and cytoplasmic degradation associated with vacuolar membrane breakdown (O’Brien and Thimann 1967, Srivastava and Singh 1972). A pivotal advancement in the study of xylogenesis was the establishment of the *Zinnia* experimental system by Fukuda and Komamine (1980), wherein mesophyll cells were induced to transdifferentiate into TEs in the presence of auxin and cytokinin (Fukuda 1994, 1992). This model facilitated the identification of cytological and molecular markers of xylogenesis and greatly enriched the understanding of PCD and its associated events, such as chromatin condensation, tonoplast rupture, and DNA fragmentation (Kuriyama 1999, Fukuda 2004, Bonneau et al. 2008, Twumasi et al. 2010, Petzold et al. 2012, Escamez and Tuominen 2014). Further microscopic and molecular studies conducted in *Populus* revealed that cytoplasmic clearance in developing TEs is mediated by autophagy, and three distinct forms of autophagy (micro-, macro-, and mega-autophagy) were identified (Bagniewska-Zadworna et al. 2012, 2014, Wojciechowska et al. 2019, Michalak et al. 2024).

The development of advanced molecular biology techniques has significantly deepened insight into the complex regulatory networks governing xylogenesis (Kułak et al. 2023). Laser microdissection and fluorescence-activated cell sorting, combined with high-throughput expression analyses, have proven instrumental in identifying novel regulators of secondary growth and xylogenesis (Marzec-Schmidt et al. 2019, Chen et al. 2021, Li et al. 2023, Tung et al. 2023). These approaches enable detailed analysis of gene expression, protein interactions, and cellular dynamics, revealing key signaling pathways, many of which are regulated by phytohormones (Sehr et al. 2010, Didi et al. 2015, Felten et al. 2018, Seyfferth et al. 2018, Behr et al. 2019, Johnsson et al. 2019, Lee et al. 2019, Tang et al. 2020, Liu et al. 2021, Zhao et al. 2023). However, despite significant recent advances, fully elucidating the roles of individual phytohormones remains challenging due to the intricate hormonal crosstalk that orchestrates the regulatory cascades underlying xylogenesis. A deeper understanding of these molecular mechanisms is critical for advancing efforts to improve plant growth and wood quality.

Among the diverse regulators of xylogenesis, phytohormones play a pivotal role in integrating developmental and environmental signals to coordinate xylem differentiation. Notably, jasmonic acid (JA) and its derivatives (jasmonates, JAs) have recently gained attention as key modulators of vascular tissue formation, alongside auxins (Xu et al. 2019, Tang et al. 2020), cytokinins (Nieminen et al. 2008, Qi et al. 2020), and abscisic acid (Liu et al. 2021). JA biosynthesis begins in plastids, where linolenic acid is converted into 12-oxo-phytodienoic acid (OPDA) through reactions catalyzed by lipoxygenase, allene oxide synthase, and allene oxide cyclase (Heitz et al. 2016, Huang et al. 2017, Heitz et al. 2019). OPDA is subsequently transported to the peroxisome, where it is reduced by OPDA reductase and further processed through β-oxidation to yield the biologically relevant stereoisomer (+)-7-iso-JA (Schaller and Stintzi 2009). JA can then be converted into a variety of active, inactive, or partially active derivatives through different metabolic pathways. Among these, JA conjugation to isoleucine is of particular importance, as jasmonoyl–isoleucine (JA-Ile) specifically interacts with the jasmonate receptor CORONATINE INSENSITIVE 1, triggering ubiquitin-mediated degradation of JAZ repressors via the 26S proteasome and activating JA-responsive genes (Ruan et al. 2019). The levels of various JA forms are tightly regulated by several enzymes, in particular three catabolic pathways characterized in *Arabidopsis* that attenuate JA-Ile signaling (Heitz et al. 2019). Firstly, CYTOCHROME P450 enzymes (CYP94B3 and CYP94B1) oxidize JA-Ile to form the less active derivative 12-OH-JA-Ile (Kitaoka et al. 2011, Heitz et al. 2012, Koo et al. 2014) and CYP94C1 produces the inactive derivative 12COOH-JA-Ile. The second pathway consists in two AMIDOHYDROLASES (IAR3 and ILL6) that cleave the isoleucine group from JA-Ile and 12-OH-JA-Ile, yielding JA and 12-OH-JA, respectively (Widemann et al. 2013). Finally, 2-OXOGLUTARATE OXYGENASES hydroxylate JA to 12-OH-JA, preventing its activation into JA-Ile, and hold a regulatory function by modulating basal defense responses (Caarls et al. 2017, Smirnova et al. 2017, Marquis et al. 2022, Ndecky et al. 2025).

The present study was directed toward identifying key regulators involved in poplar (*Populus trichocarpa*) stem and pioneer root development, with particular emphasis on secondary growth formation and the process of xylogenesis. Through the application of high-throughput techniques, a set of genes exhibiting significantly altered expression during these developmental processes was identified (Marzec-Schmidt et al. 2019, 2020). Among the numerous candidates, genes encoding 2-OXOGLUTARATE (2-OG) AND FE(II)-DEPENDENT OXYGENASES were notably upregulated during secondary growth in both examined organs. Despite their induction, the specific functions of the proteins encoded by these genes remain uncharacterized, particularly within the context of xylogenesis. Clarifying the functional roles of these enzymes is essential for advancing the understanding of the molecular regulation of secondary growth in *P. trichocarpa*, with potential implications for enhancing wood development, quality, and sustainability in both forestry and agricultural applications. Considering that wood is a natural alternative to synthetic materials, increasing wood utilization is necessary to implement strategies mitigating climate change (Demura and Fukuda 2007). To determine the roles of the target oxygenases in xylogenesis, part of the investigation was conducted using *Arabidopsis*, owing to the immediate availability of mutant lines in orthologous genes. Furthermore, performing parallel analyses in two taxonomically distinct species offers a broader framework for interpreting the functional significance of these proteins and supports the hypothesis that their roles are conserved across plant lineages.

## Results

### Expression of 2-OG- and Fe(II)-dependent oxygenases during stem and pioneer root development

**Figure 1:**
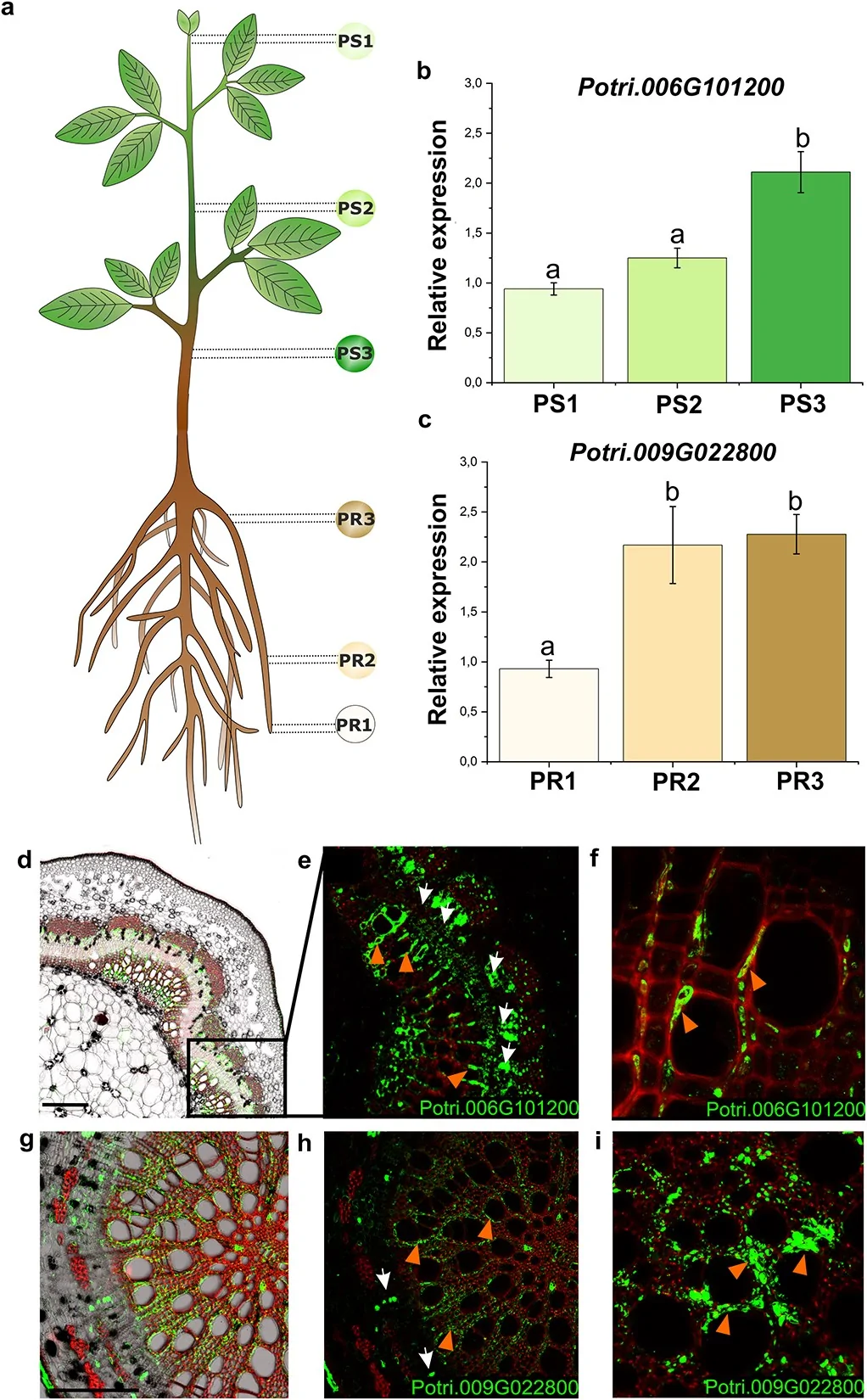
Analysis of gene expression encoding 2-OG oxygenases in the stems and pioneer roots of *Populus trichocarpa*. (a) Developmental stages selected for analysis: PS1—apical meristem with primary development; PS2—early secondary growth; PS3—mechanically isolated secondary xylem; PR1— root tips with apical meristem; PR2—primary development; PR3—secondary development. (b, c) Relative expression of *Potri.006G101200* and *Potri.009G022800* across different stages of stem and root development in *P. trichocarpa*. Means labeled with the same letter do not differ significantly according to Tukey’s *post hoc* test (*P* < 0.05 ± SE). (d–f) Localization of *Potri.006G101200* mRNA in stems. (g–i) Localization of *Potri.009G022800* mRNA pioneer roots. Lignin autofluorescence is shown in red. Bars: 200 *μ*m.

A poplar microarray was used to identify gene expression changes during stem and pioneer root development (Fig. 1a). The analyses were conducted on organ fragments corresponding to specific developmental stages, as confirmed by anatomical studies (Supplementary Figure S1). Among the differentially expressed genes (DEGs), two genes (*Potri.006G101200* in stems and *Potri.009G022800* in pioneer roots) were identified as encoding 2-OXOGLUTARATE AND FE(II)-DEPENDENT OXYGENASES (2-OG oxygenases), exhibiting increased expression during the later stages of organ development. The expression of *Potri.006G101200* showed a 73-fold increase in mature xylem (PS3) compared with the primary developmental stage (PS1), whereas *Potri.009G022800* exhibited an ~5-fold higher expression in both the primary (PR2) and secondary (PR3) developmental stages relative to the root meristem. Results from microarray analyses were validated by RT-qPCR, which confirmed these expression trends (Fig. 1b and c).

To determine the tissue-specific expression patterns of the identified genes, fluorescence *in situ* hybridization (FISH) was performed. *Potri.006G101200* mRNA localized to the secondary vascular tissues of the stem, including both the phloem (Fig. 1e, white arrows) and xylem (Fig. 1e and f, orange arrowheads). Within the secondary xylem, the signal intensity was the strongest in parenchyma cells surrounding the vessels (Fig. 1d–f). Similarly, *Potri.009G022800* mRNA in pioneer roots was primarily detected in the secondary xylem (Fig. 1h–i, orange arrowhead), with an additional strong punctate signal observed in the secondary phloem (Fig. 1h, white arrow). The signal distribution closely resembled that in stems, with fluorescence primarily concentrated in small parenchyma cells adjacent to vessels (Fig. 1i). These findings indicate that the upregulation of 2-OG oxygenase genes is associated with vascular tissue development, particularly within the secondary xylem.

### Analysis of potential orthologs of the poplar oxygenase genes and AI protein structure prediction

The precise roles of *Potri.006G101200* and *Potri.009G022800* in secondary xylem formation remain unclear. To gain functional insights, potential orthologs were sought in the *Arabidopsis* genome using the JGI Phytozome database (Supplementary Table S1). A literature search and sequence similarity analyses identified four *Arabidopsis* genes—*JAO1/JOX1* (*AT3G11180*), *JAO2/JOX2* (*AT5G05600*), *JAO3/JOX3* (*AT3G55970*), and *JAO4/JOX4* (*AT2G38240*)—annotated as JA OXYGENASES (JAOs) or JASMONATE-INDUCED OXYGENASES (JOXs) (Caarls et al. 2017, Smirnova et al. 2017). These showed high sequence similarity to the poplar 2-OG oxygenases, particularly with *Potri.006G101200* (Supplementary Figure S2). Phylogenetic analysis further supported this relationship, showing that *Potri.006G101200* clusters closely with its *Arabidopsis* orthologs as well as with other proteins annotated as JAO from *Oryza sativa* and *Nicotiana attenuata*, whereas *Potri.009G022800* appears to be phylogenetically more divergent (Supplementary Figures S3 and S4).

**Figure 2:**
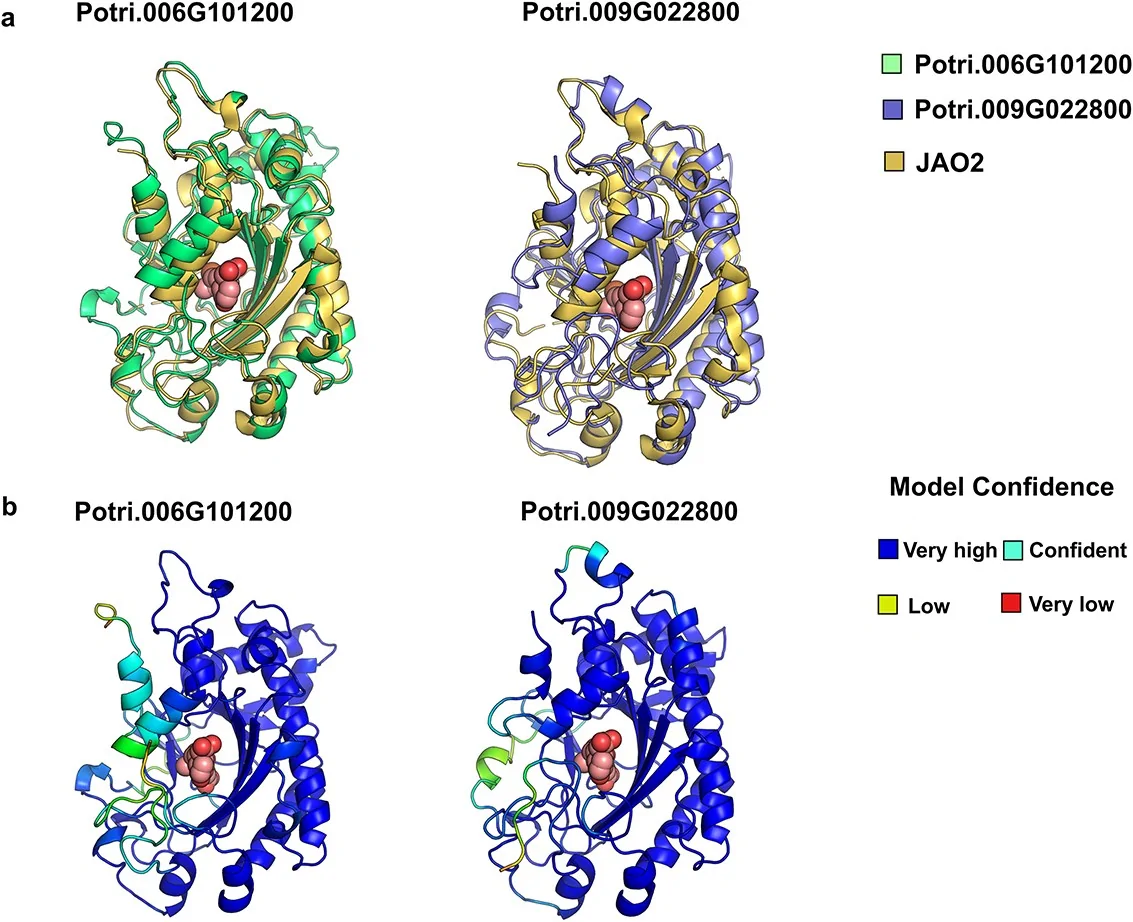
Structural overview of potential JA oxidases. (a) Structural superposition of each potential oxygenase identified from microarray analyses with the JAO2/JOX2 structure (PDB code: 6LSV) used as a reference. The orientation of the JA ligand within the catalytic pocket of the oxygenase is highlighted. (b) Predicted JA oxidases colored according to the pLDDT model confidence scores, indicating the precision of the obtained structural models.

To further investigate these enzymes, protein structure models for the poplar oxygenases were generated using AlphaFold2 (AF2) (Jumper et al. 2021). Although Potri.009G022800 shares only ~55% sequence similarity with JAO2/JOX2, its notable structural resemblance to JAO2 and Potri.006G101200 prompted us to include both enzymes in subsequent analyses aimed at exploring their potential involvement in JA catabolism. The structural models of *Populus* proteins showed high to very high confidence scores (pLDDT > 80), indicating high prediction accuracy (Fig. 2a and b). However, reduced model confidence was observed at the N- and C-terminal regions and in a loop region proximal to the putative JA binding site (Fig. 2b), suggesting that these regions may be structurally flexible. Importantly, the core structural elements of the predicted models were highly similar to those of *Arabidopsis* JAO2/JOX2, particularly in terms of secondary structure (Zhang et al. 2021). Root mean square deviation (RMSD) values indicated close structural alignment, with values of 1.36 Å for *Potri.006G101200* and 1.75 Å for *Potri.009G022800*. These data underscore the structural conservation of the functional domains and support the hypothesis that these *Populus* proteins may function as JAOs.

### Validation of docking method

**Figure 3:**
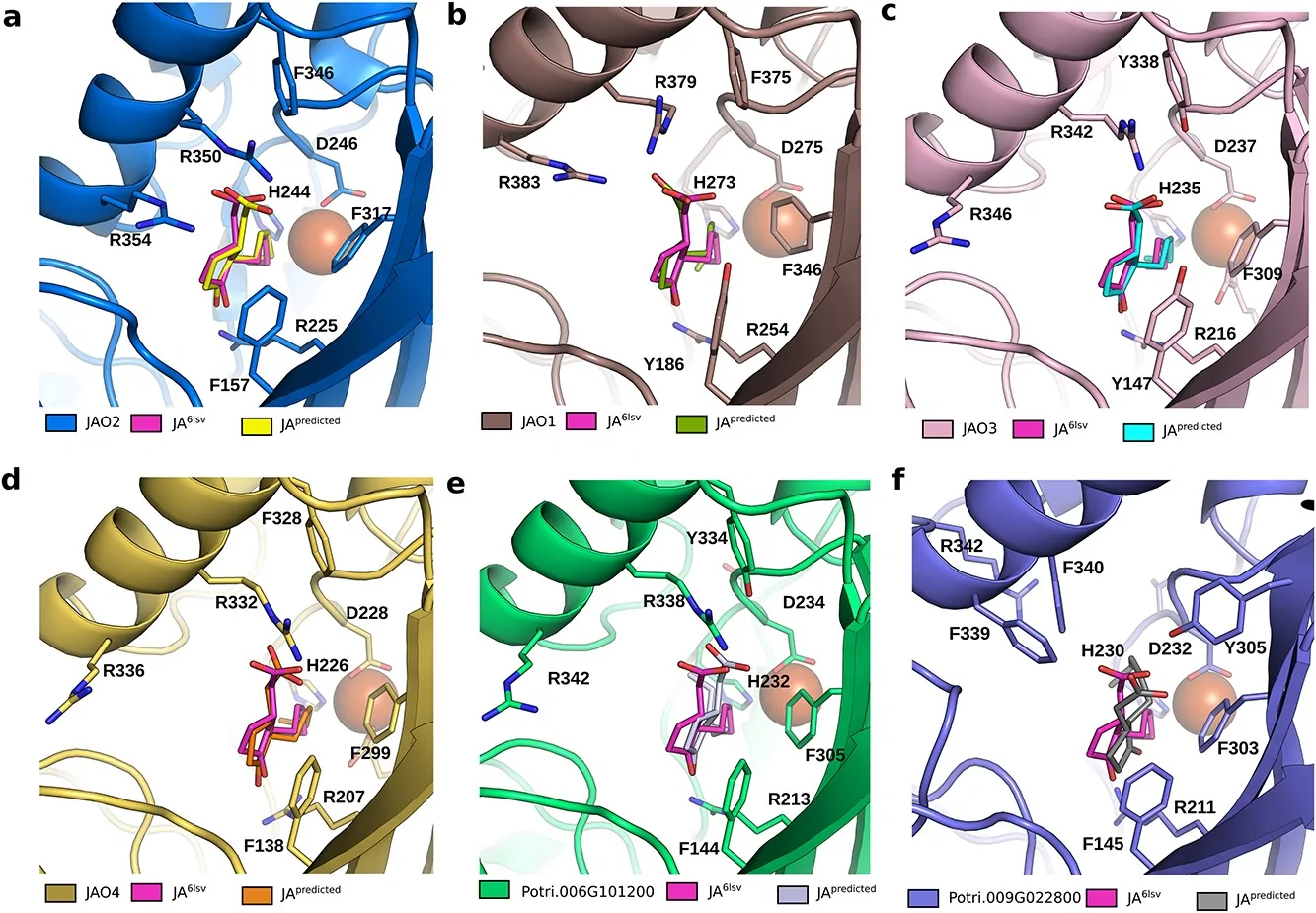
Docking studies of JA ligand. (a) Molecular docking of the JA ligand to the JAO2/JOX2 protein structure. The best docking pose of JA is shown in comparison with its reference orientation in the crystal structure (PDB code: 6LSV). (b) Molecular docking of the JA ligand to the JAO1/JOX1 protein. The best docking orientation is shown in comparison with the reference ligand JA from 6LSV. (c) Molecular docking of the JA ligand to the JAO3/JOX3 protein. The best docking orientation is shown in comparison with the reference ligand JA from 6LSV. (d) Molecular docking of the JA ligand to the JAO4/JOX4 protein. The best docking orientation is shown in comparison with the reference ligand JA from 6LSV. (e) Molecular docking of the JA ligand to the Potri.006G101200 protein. The best docking orientation is shown in comparison with the reference ligand JA from 6LSV. (f) Molecular docking of the JA ligand to the Potri.009G022800 protein. The best docking orientation is shown in comparison with the reference JA ligand from 6LSV. Crucial residues involved in binding are labeled, and the iron ion is represented as an orange sphere.

Prior to conducting molecular docking simulations, the docking algorithm was validated using the crystal structure of the JAO2/JOX2 protein complexed with JA (PDB: 6LSV) (Zhang et al. 2021). Specifically, docking simulations of the JA ligand were performed using the Induced Fit Docking (IFD) protocol. The resulting docking poses closely matched the experimental configuration (Fig. 3a), with the highest-scoring pose accurately reproducing the orientation of JA in the 6LSV crystal structure. The root-mean-square deviation (RMSD) for the ligand position was 0.35 Å relative to the experimental JA orientation (calculated for nonhydrogen atoms only).

### Docking studies of potential JAOs

To evaluate the potential of JA binding to the selected *Populus* proteins, molecular docking simulations were performed using the IFD protocol. This protocol was selected because regions surrounding the predicted JA binding site were identified as potentially flexible, based on low confidence scores in AF2 structural predictions (Fig. 2b).

Docking analyses showed favorable JA binding for both Potri.006G101200 and Potri.009G022800, with affinities comparable to *Arabidopsis* JAO2/JOX2 (Table 1 and Fig. 3b–f). The predicted binding poses displayed low RMSD values (1.1 and 1.6 Å, respectively), indicating slightly weaker binding for Potri.009G022800 (Table 1). Structural inspection revealed that Potri.006G101200 retains the canonical arginine triad (R213, R338, and R342) and key aromatic residues (F144, F305, and Y334) required for JA recognition, whereas Potri.009G022800 lacks the full arginine triad but preserves the aromatic residue arrangement (F145, F303, and Y305). Molecular dynamics simulations confirmed stable JA binding in both proteins. Overall, Potri.006G101200 emerges as a strong candidate JAO, while the results for Potri.009G022800 are less conclusive but do not exclude its potential JAO activity; therefore, both proteins were subjected to experimental validation.

**Table 1:** Docking score, binding energy, RMSD values, and MD simulation data determined from molecular docking and MD simulations for potential JA oxidases and JAO2/JOX2 from *A. thaliana* as the reference structure.

**Protein name**	**Docking score** ^**a**^	**RMSD** ^**b**^	**E bind** ^**c**^	**MD data** ^**d**^
**Potri.006G101200**	−5.23	1.1	−32.15	Stable
**Potri.009G022800**	−5.44	1.59	−38.33	Stable
**AtJAO2**	−6.59	0.35	−43.33	Stable
**AtJAO1**	−4.84	0.56	−18.97	Stable
**AtJAO3**	−3.99	0.42	−22.81	Stable
**AtJAO4**	−4.87	0.79	−32.47	Stable

a Docking score was obtained using the IFD molecular docking protocol.

b RMSD for the JA docking pose was calculated over nonhydrogen atoms of the JA ligand relative to its orientation in the PDB 6LSV structure.

c MM-GBSA binding energy estimation was evaluated using the Prime module.

d Based on MD simulation data, the ligand pose was classified as stable or unstable within the catalytic pocket of the potential oxidases.

### Enzymatic assays of potential *Populus* JAOs

**Figure 4:**
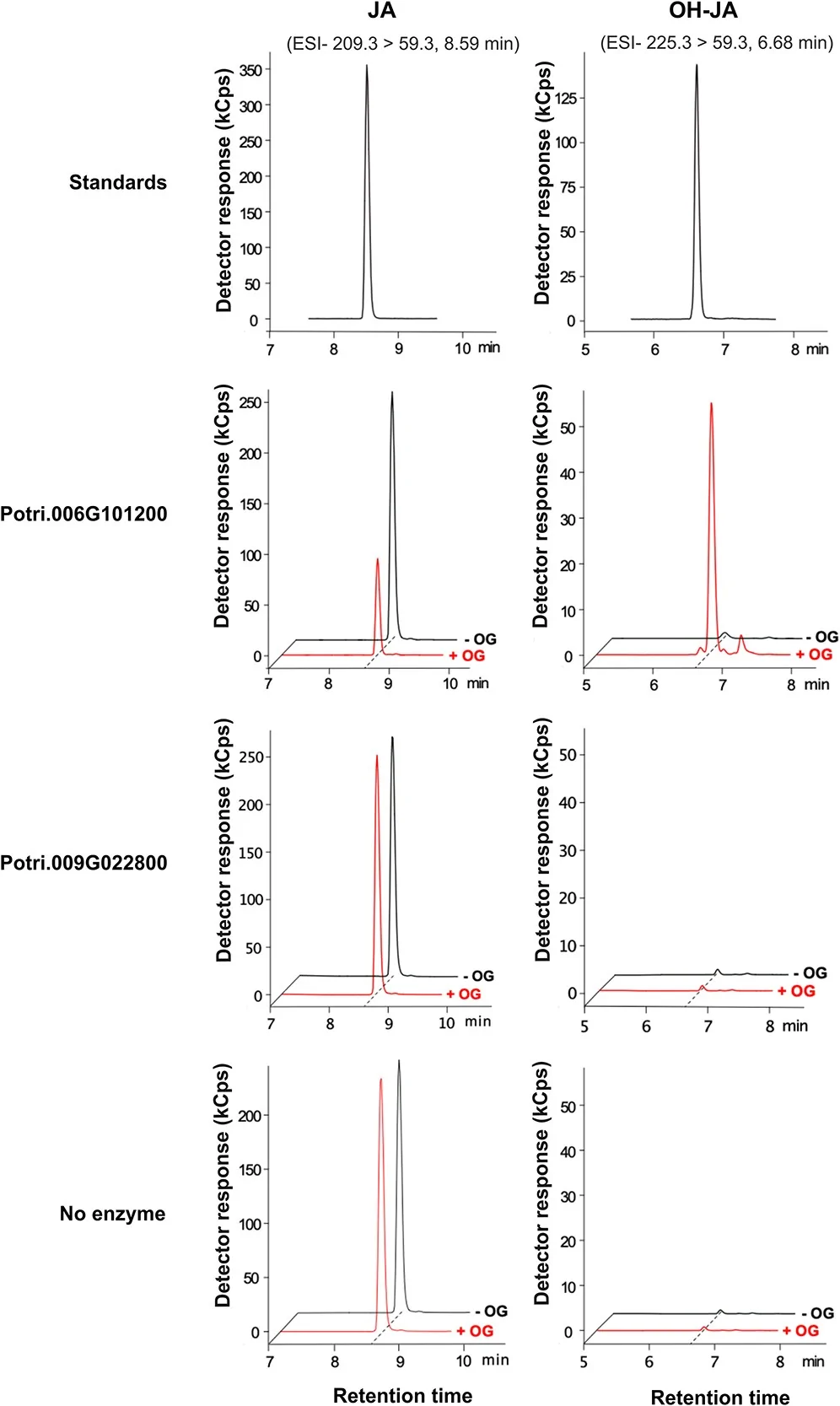
JA oxidase activity assay of recombinant Potri.006G101200 and Potri.009G022800 proteins. Proteins were expressed in bacteria and affinity-purified. Twenty micrograms of candidate proteins were incubated with 50 *μ*M JA substrate in 200 *μ*L reactions for 1 h at 28°C. Control reactions (-OG, black traces) were carried out in the absence of the studied proteins (no enzyme) or 2-OG cosubstrate. The oxidation product generated by Potri.006G101200 (+OG, red trace) exhibited retention time and mass transitions matching the authentic OH-JA standard. ESI-, Electrospray ionization, negative mode; kCps, kilocounts.

To determine directly whether the selected *Populus* 2-OG oxygenases can catalyze the conversion of JA to hydroxy-JA (OH-JA), the enzymes were expressed as recombinant proteins in *Escherichia coli* and subsequently affinity-purified. Enzymatic assays were conducted using JA as the substrate. Considering that 2-OG oxygenases typically catalyze hydroxylation reactions using 2-OG as a cosubstrate, an additional control reaction lacking 2-OG was performed. The results indicated that only Potri.006G101200 consumed readily the JA substrate and generated a product matching the retention time and mass transition of an authentic OH-JA reference (Fig. 4). The reaction was 2-OG-dependent, and this result therefore qualifies Potri.006G101200 as a poplar JA oxidase.

### Exogenous induction of oxygenase gene expression

**Figure 5:**
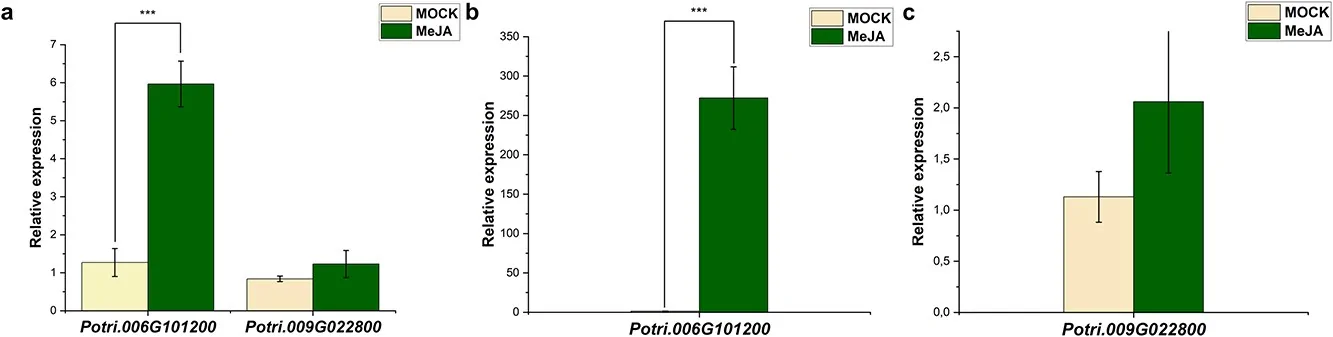
The relative expression of *Potri.009G022800* and *Potri.006G101200* following MeJA treatment in the leaves (a), roots (b), and stems (c) of *P. trichocarpa*. Leaves were selected for analysis because of their large surface area, allowing a stronger response to MeJA treatment. To confirm that the expression of *Potri.006G101200* and *Potri.009G022800* was not organ-specific, the genes were also analyzed in stems and roots, where microarray analyses had previously shown increased expression. The error bars indicate standard error. The asterisks represent statistical significance based on Student’s *t*-test between mock and MeJA-treated samples. A single asterisk (*) indicates a statistically significant *P*-value, and three asterisks (***) denote a *P*-value < 0.0001.

To evaluate a potential link of the two studied poplar 2-OG oxygenases with jasmonate regulation, we examined their transcriptional response to methyl jasmonate (MeJA) exposure. *Populus* seedlings were treated with MeJA, and the expression of *Potri.006G101200* and *Potri.009G022800* was analyzed in three distinct organs: leaves, stems, and roots. Leaves were selected due to their large surface area, offering the greatest potential for responsiveness to MeJA treatment (Fig. 5a). Stems and roots were included to relate the findings to previously studied organs characterized by more extensive xylogenesis. The results demonstrated that expression of *Potri.006G101200* was significantly induced by MeJA in both leaves and stems (Fig. 5a and c). While a slight increase in expression of *Potri.009G022800* was observed, the changes were not statistically significant in either of the examined organs (Fig. 5a and b). The results, together with those from enzyme assays, indicate that only *Potri.006G101200* encodes a JA-responsive JA oxidase and raise the question of a potential link of JA hydroxylation with xylogenesis.

### Localization of JA in *Populus* stem

Following confirmation that *Potri.006G101200* encodes a JAO, the spatial distribution of JA during *Populus* stem development was examined. Immunolocalization analyses revealed the presence of JA in cortical parenchyma cells as well as in vascular bundles during primary stem growth (Supplementary Material S6a–c). During secondary growth, a strong fluorescence signal was predominantly observed in vascular tissues, particularly within the secondary xylem (Supplementary Figure S5d). JA was detected in differentiating xylem cells prior to PCD, as well as in xylem parenchyma cells (Supplementary Figure S5e and f, arrowheads). This distribution pattern closely corresponded to the localization of *Potri.006G101200* mRNA transcripts observed previously (Fig. 1f). The presence of JA and the *JAO* transcripts in overlapping regions of the secondary xylem suggests that maintaining proper JA levels through controlled catabolism is critical for the proper progression of xylogenesis.

### Analysis of potential orthologs of the poplar oxygenase genes during inflorescence stem and root development in *Arabidopsis thaliana*

**Figure 6:**
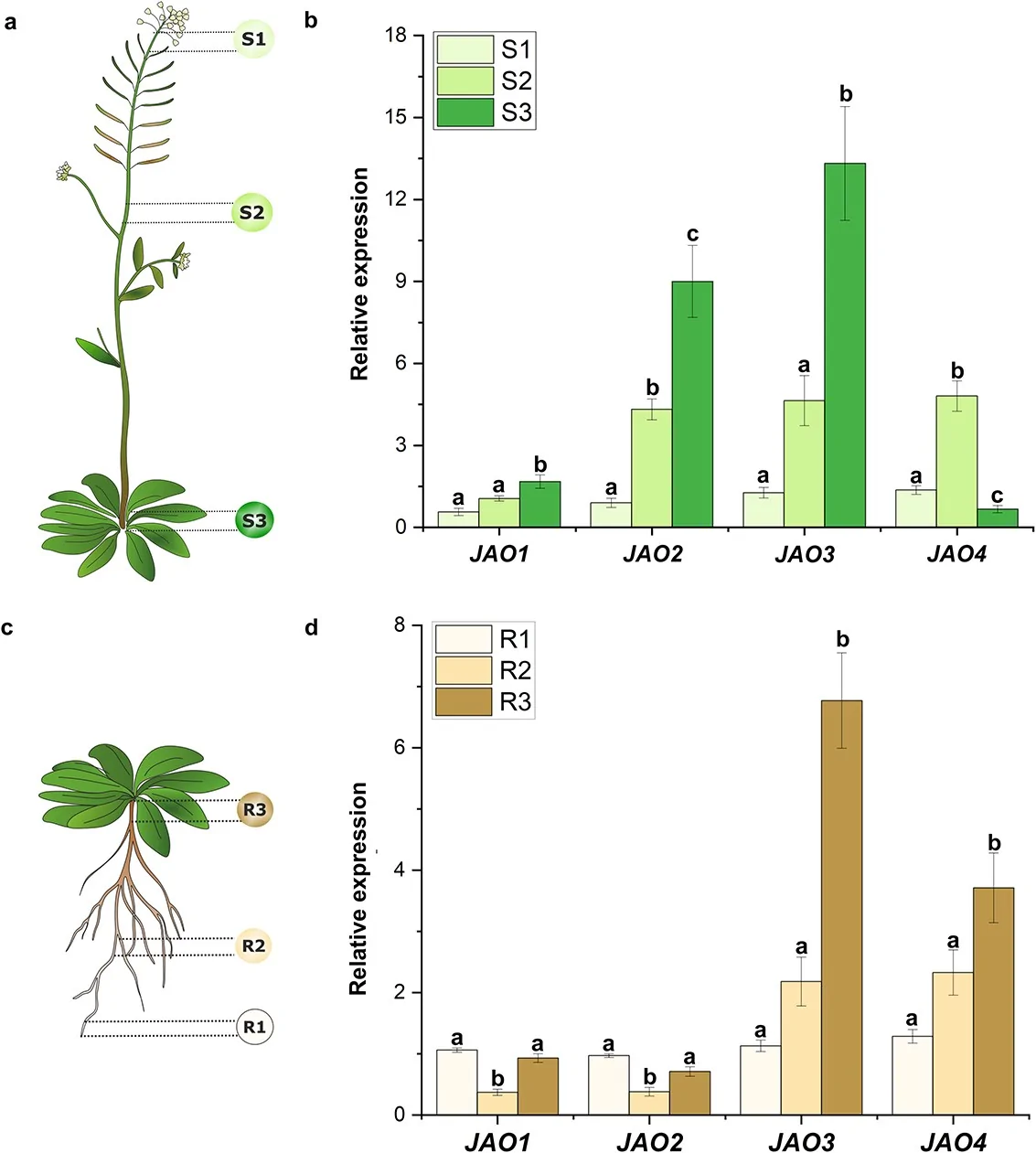
Relative expression of *JAO* genes across developmental stages (a, c) of inflorescence stem (b) and roots (d) in *A. thaliana* (WT). The error bars indicate standard error. Means labeled with the same letter do not differ significantly (Tukey’s *post hoc* test, *P* < 0.05 ± SE). S1: 0–0.5 cm—apical meristem with stem primary development; S2—Stem early secondary growth; S3—advanced stem secondary growth; R1—root tips with apical meristem; R2—primary root development; R3—secondary root development.

Given the current lack of genetic tools to track the function of JAO activity in poplar, we examined expression of the four described *AtJAO* genes (Smirnova et al. 2017) in a xylogenesis context in *Arabidopsis*. Expression analysis was conducted across three developmental stages of stem and root as sites of xylogenesis (Fig. 6a and c; Supplementary Figure S1). In the inflorescence stem, *AtJAO1*, *AtJAO2*, and *AtJAO3* displayed a progressive increase in expression, with maximal levels during advanced secondary growth (S3), while *AtJAO4* peaked earlier at S2 (Fig. 6b). In roots, expression levels of *AtJAO3* and *AtJAO4* increased during secondary development (Fig. 6d). This expression pattern parallels that of the *Populus* 2-OG oxygenase genes (Fig. 1b and c), suggesting a possible role for AtJAOs in regulating secondary growth processes.

### The influence of mutations in *JAO*/*JOX* genes on the anatomical structure of the inflorescence stem in *A. thaliana*

**Figure 7:**
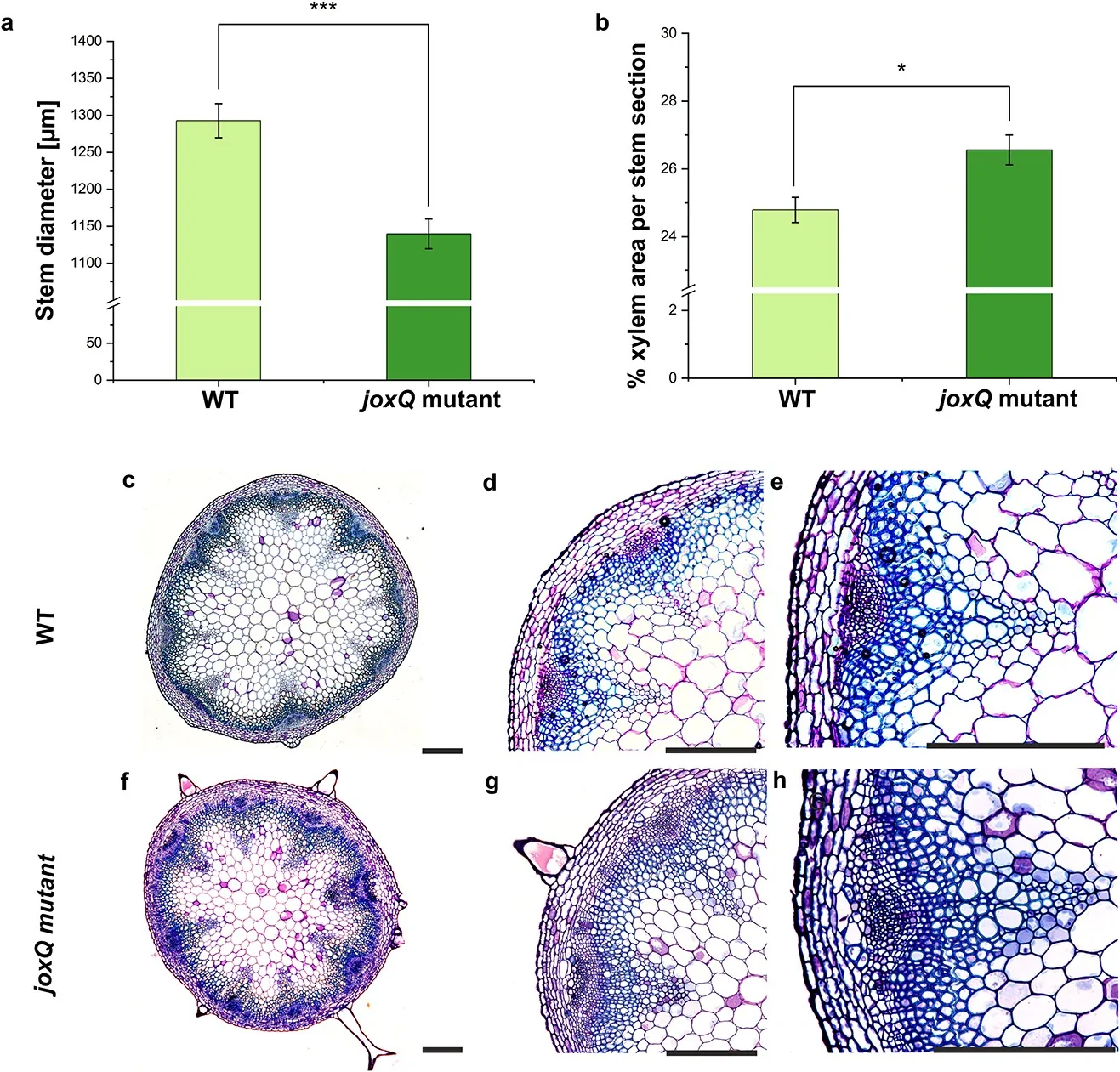
Comparison of inflorescence stem anatomy between WT and *joxQ* mutant of *A. thaliana*. (a–c) Changes in stem diameter between the WT and the *joxQ* mutant; (b) Comparison of the xylem area relative to the total cross-sectional area of the stem, expressed as a percentage. (c–h) *Arabidopsis thaliana* stem sections at S3 stage were collected, fixed, and stained with toluidine blue before observation under a microscope. (c–e) Anatomical structure of the inflorescence stem in the WT. (f–h) Anatomical structure of the inflorescence stem in the *joxQ* mutant. The error bars in (a, b) indicate standard error. The asterisks denote statistical significance based on Student’s *t*-test comparing the WT and *joxQ*. A single asterisk (*) indicates *P* < 0.05, while three asterisks (***) indicate *P* < 0.0001. Scale bars = 200 *μ*m.

To assess whether the absence of *JAO*/*JOX* gene expression affects the anatomical structure of the inflorescence stem, a quadruple knockout mutant line of *JAO*/*JOX* genes (*joxQ*) was analyzed. This mutant was characterized previously by impaired hydroxylation of JA and subsequent increased accumulation of JA in leaves (Caarls et al. 2017). Morphological and anatomical analyses revealed that the *joxQ* mutant exhibited a reduced stem diameter compared to the wild type (WT) (Fig. 7a). Notably, despite the decrease in overall stem diameter and total cross-sectional area, the proportion of xylem within the stem was higher in *joxQ* (Fig. 7b and f–h) than in WT plants (Fig. 7b and c–e). These results suggest that JA accumulation, due to the loss of *JAO*/*JOX* activity, may be associated with reduced plant size while concomitantly promoting xylem development. This finding highlights the critical role of *JOX* enzymes in regulating xylogenesis.

## Discussion

The development of vascular tissues was a key evolutionary innovation that enabled plants to colonize terrestrial environments (Li et al. 2010). Although vascular development has been extensively studied, its regulatory mechanisms remain only partially understood. Xylogenesis involves a coordinated sequence of events: cambial activity, SCW formation, lignification, and programmed cell death, resulting in functional, hollow conduits for long-distance transport (Wood 2002, Samuels et al. 2006). Precise regulation of these processes is therefore essential for proper xylem function, and such stringent regulatory demands point to the involvement of a highly coordinated and multilayered molecular framework.

The complex molecular network governing secondary xylem development has been the subject of numerous studies (Peter and Neale 2004, Schuetz et al. 2013, Růžička et al. 2015, Yang and Wang 2016, Marzec-Schmidt et al. 2019, 2020, Tang et al. 2020, Zhang et al. 2020). It has been demonstrated that the expression of hundreds of genes is altered during secondary growth (Hertzberg et al. 2001, Marzec-Schmidt et al. 2019, Chen et al. 2021). These genes can be categorized into functional groups associated with specific stages of xylogenesis, including SCW formation (Hertzberg et al. 2001, Karlsson et al. 2005, Song et al. 2010, Marzec-Schmidt et al. 2019), lignification (Hertzberg et al. 2001, Chen et al. 2021, Li et al. 2023), and PCD (Funk et al. 2002, Wojciechowska et al. 2019, Li et al. 2023).

In high-throughput gene expression analyses of *P. trichocarpa* stem and pioneer root development, a significant upregulation was observed for two genes, *Potri.006G101200* and *Potri.009G022800*, which encode 2-OG- AND FE(II)-DEPENDENT OXYGENASES. However, the precise role of these proteins in xylogenesis remains unclear. The 2-OG oxygenases constitute a large superfamily present across bacteria, fungi, plants, and vertebrates (Jia et al. 2017). In plants, they represent the second largest enzyme family (Kawai et al. 2014), with broad functional roles, including protein modification, fatty acid metabolism, carnitine biosynthesis, phytanic acid catabolism, and nucleic acid repair, regulation, and modification (Farrow and Facchini 2014, Jia et al. 2017). Importantly, 2-OG oxygenases are also central to phytohormone metabolism, with confirmed involvement in the biosynthesis of gibberellins (GAs) (Lange et al. 1994) and the catabolism of auxin (Zhao et al. 2013, Porco et al. 2016), salicylic acid (Zhang et al. 2013), and GAs (Schomburg et al. 2003, Lee and Zeevaart 2005, Rieu et al. 2008). Among the *Populus* 2-OG oxygenases identified in this study, Potri.006G101200 exhibited a high degree of both sequence and structural similarity to the *Arabidopsis* enzymes annotated as JAOs (Smirnova et al. 2017) or JOXs (Caarls et al. 2017), which are known to play key roles in JA metabolism. In contrast, Potri.009G022800, despite sharing only ~55% sequence identity, showed substantial structural resemblance to its *Arabidopsis* homologs, suggesting potential conservation of catalytic function.

Studies have demonstrated that expression of *JAO*/*JOX* genes is induced in response to JA (Caarls et al. 2017), and multiple investigations have emphasized the importance of these enzymes in regulating responses to both biotic and abiotic stress (Smirnova et al. 2017, Marquis et al. 2022). An *Arabidopsis joxQ* knockout mutant, lacking the four *JAO*/*JOX* genes, exhibited phenotypes consistent with heightened JA responses, including increased resistance to the necrotrophic fungus *Botrytis cinerea* and the herbivorous insect *Mamestra brassicae*, as well as elevated expression of JA-dependent defense-related genes (Caarls et al. 2017). Interestingly, the *jao2/jox2* single mutation is sufficient to trigger increased basal defenses, improved fungal resistance, and survival under severe drought conditions (Smirnova et al. 2017, Marquis et al. 2022). However, the role of JAO/JOX proteins in developmental processes remains largely uncharacterized. Expression analysis of the four *JAO*/*JOX* genes during *Arabidopsis* inflorescence stem and root development revealed that some displayed increasing expression patterns similar to those of the studied 2-OG oxygenases in *Populus*. This suggests that the proteins encoded by *Potri.006G101200* and *Potri.009G022800* may participate in JA catabolism, and that proper regulation of JA metabolism is essential for optimal plant secondary growth, particularly in xylem formation. The *Populus* 2-OG oxygenases *Potri.006G101200* and *Potri.009G022800* were tested for their potential to bind and hydroxylate JA. Structural analyses, including molecular docking and molecular dynamics simulations, suggested the capacity of both proteins to bind JA. Notably, the calculated binding energy values were comparable to those observed for JA binding to the JAO2/JOX2 protein based on the crystal structure (Zhang et al. 2021). For both proteins, a high degree of similarity was observed in the orientation of the cyclopentane ring and the carboxyl-terminated side chain relative to the JA binding pose in 6LSV. However, the carbon chain containing double bonds exhibited a distinct orientation compared to the JA configuration in the reference structure. Examination of the JA binding sites in *Potri.006G101200* and *Potri.009G022800* revealed a key difference in amino acid composition critical for JA binding and hydroxylation. As shown by Zhang et al. (2021), an arginine triad plays a central role in JA recognition and catalysis in *Arabidopsis* JAO2/JOX2. This arginine triad is present in *Potri.006G101200*, but absent in *Potri.009G022800*, where one arginine is substituted by phenylalanine. Although phenylalanine can still interact with the JA molecule, its binding is less favorable compared to arginine. These *in silico* results align with *in vitro* and *in vivo* findings, where only *Potri.006G101200* expression was significantly induced following MeJA treatment. These results support the hypothesis that Potri.006G101200 may function in the conversion of JA to OH-JA during secondary growth formation and xylogenesis.

The observed upregulation of 2-OG oxygenases in both *Arabidopsis* and *Populus* tissues undergoing xylogenesis may thus be associated with the regulation of jasmonate homeostasis, maintaining appropriate levels of active and inactive JA derivatives throughout the stages of secondary growth. The involvement of JA in xylogenesis is well documented, spanning early events such as cambial activation to later stages of xylem differentiation. Notably, exogenous MeJA application has been shown to stimulate cambial cell division (Sehr et al. 2010), which may explain why an increased xylem area was observed in the *joxQ* mutant that accumulates JA. Similar findings have been reported in *Arabidopsis* mutants hypersensitive to JA, such as *jaz10-1*, which display enhanced lateral extension of intervascular cambium-derived tissues and increased stem diameter (Sehr et al. 2010). In contrast, the *joxQ* mutant in this study showed reduced stem diameter, consistent with earlier observations (Caarls et al. 2017) that loss of JAO/JOX activity negatively impacts overall plant growth and seed production. Interestingly, despite the reduced stem size, the proportion of xylem relative to the total stem area was larger in *joxQ* than in the WT.

An increasing number of studies highlight the role of JA in SCW formation and lignification (Behr et al. 2019, Zhao et al. 2023, Im et al. 2024). MeJA treatment has been shown to promote lignin deposition and SCW thickening in *Arabidopsis* (Sehr et al. 2010), and mechanical injury is known to elevate JA levels and stimulate lignin biosynthesis (Denness et al. 2011). Zhao et al. (2023) recently demonstrated that MeJA application enhances expression of SCW biosynthetic genes in poplar, promoting lignification and wall thickening. Conversely, reduced JA levels in *Populus* led to SCW thinning, as observed in the *opdat1* mutant, an effect reversed by JA treatment. These responses are partially mediated by JAZ5, a repressor of JA signaling that interacts with SCW transcriptional regulators such as WND6A and MYB3, inhibiting activation of SCW-related genes (Zhao et al. 2023). Additional work in *A. thaliana* has identified a central JA signaling regulator (MYC2) as a transcriptional activator of *MYB46*, a master regulator of SCW synthesis. The MYC2–MYB46 regulatory module has been proposed as a critical mechanism linking JA signaling to SCW formation (Im et al. 2024). Interestingly, studies in *Cannabis sativa* hypocotyls reported no JA-related changes in xylem development, as expression of xylem-associated genes remained unaffected following MeJA treatment (Behr et al. 2019). However, these studies highlighted a prominent role for JA in promoting phloem fiber development. Similar conclusions were reached by Sehr et al. (2010), who observed increased fiber cell formation in *Arabidopsis* stems following JA treatment.

In conclusion, the findings demonstrate that 2-OG oxygenases constitute key components of the molecular machinery governing secondary growth, playing a particularly prominent role in the regulation of xylogenesis. Bioinformatic analyses, supported by *in vitro* and *in vivo* validation, identified *Potri.006G101200* as a JA OXIDASE involved in maintaining the balance between active and inactive JA derivatives during secondary growth. Functional studies using *Arabidopsis* mutants elucidated the contribution of *JAO*/*JOX* genes to the xylogenesis process. Anatomical analyses of the *joxQ* mutant revealed that disruptions in JA catabolism, resulting from the absence of JAO/JOX activity, may enhance cambial cell division. These findings suggest that JAO/JOX proteins may be important players in xylogenesis and warrant further investigation. The specific molecular mechanisms by which these enzymes modulate cambial activity and secondary growth remain to be fully elucidated. Future studies integrating transcriptomic, proteomic, and metabolomic approaches will be essential for uncovering the broader roles of JAO/JOX in vascular tissue development. A deeper understanding of these regulatory networks may provide important insights into the mechanisms underlying plant growth regulation and adaptation to environmental stress.

## Materials and Methods

### Plant material and growth condition

*Populus trichocarpa* and *A. thaliana* were used in the experiments. *P. trichocarpa* seedlings were initially cultivated for 3 months in a plant growth chamber (Conviron GR96, Winnipeg, MB, Canada) under controlled conditions of 18°C during the day and 14°C at night, with a photoperiod of 16 h of light and 8 h of darkness. After this period, the plants were transferred to a rhizotron system at an experimental field site located at the Institute of Dendrology, Polish Academy of Sciences, in Kórnik (52°14′40″N, 17°06′27″E), as described by Wojciechowska et al. (2018). Following 2 months of growth in rhizotrons, the selected plant organs were divided into three segments corresponding to distinct developmental stages, as outlined in Table 2 and Supplementary Figure S1.

**Table 2:** Description of selected stages of stems and roots selected for analyses.

	***Populus trichocarpa***		***Arabidopsis thaliana***	
**STEM**	**PS1**	0–2 cm—apical meristem with primary development	**S1**	0–0.5 cm—apical meristem with primary development
	**PS2**	20–25 cm—early secondary growth	**S2**	12–13 cm—early secondary growth
	**PS3**	40–45 cm—mechanical isolated secondary xylem	**S3**	21–23 cm—advanced secondary growth
**ROOTS**	**PR1**	0–2 cm—root tips with apical meristem	**R1**	0–0.7 cm—root tips with apical meristem
	**PR2**	4–6 cm—primary development	**R2**	3–4.5 cm—primary development
	**PR3**	13–16 cm—secondary development	**R3**	7.5–8.5 cm—secondary development

The bold formatting indicates the names of organs, species and developmental stages and it is used to improve the readability of the table.

*A. thaliana* plants were grown in a plant growth chamber (Sanyo, Osaka, Japan) at 21°C during the day and 19°C at night, under a 16-h light and 8-h dark photoperiod. For stem analyses, plants were cultivated on a peat-based growth medium, and inflorescence stems were harvested after 7 weeks, once fully developed. For root analyses, *A. thaliana* seedlings were grown vertically on half-strength Murashige and Skoog (MS) medium supplemented with 1% agar and 1% sucrose. Root fragments were collected after 3 weeks, at which point the onset of secondary growth was confirmed by anatomical analysis (Supplementary Figure S1). During sample collection, both stems and roots were divided into three segments according to their developmental stages and subjected to further analyses (Table 2).

In addition to WT plants, a quadruple jasmonate-induced oxygenase mutant line (*joxQ*) was used. The *joxQ* mutant was generated through genetic crosses between homozygous T-DNA insertion lines in successive generations, followed by self-fertilization to obtain homozygous lines for each target gene: *SAIL_131_D01* (*At3g11180*, *JAO1/JOX1*), *GK-870-C04* (*At5g05600*, *JAO2*/*JOX2*), *SAIL_861_E01* (*At3g55970*, *JAO3*/*JOX3*), and *SAIL_268_B05* (*At2g38240*, *JAO4*/*JOX4*) (Caarls et al. 2017). The primers used for genotyping are listed in Supplementary Table S2.

### Microarray analysis

Total RNA was isolated using the RNeasy Plant Mini Kit (Qiagen, Hilden, Germany). Complementary RNA (cRNA) synthesis and hybridization to the Affymetrix GeneChip Poplar Genome Array (A-AFFY-131, Santa Clara, CA, USA) were performed according to the Affymetrix protocol. Microarray experiments were conducted at the Laboratory of Microarray Analysis, Institute of Biochemistry and Biophysics, Polish Academy of Sciences (Warsaw, Poland). The complete dataset is available in the Gene Expression Omnibus (GEO) database (accession number GSE126842). Raw image data from three A-AFFY-131 arrays were normalized using the robust multiarray average method. Normalized data were statistically analyzed using GeneSpring GX7 13.1 software (Agilent Technologies Inc., Santa Clara, CA, USA). A one-way analysis of variance (ANOVA) was applied with a Benjamini–Hochberg-corrected *P*-value cutoff of 0.05. Analyses were restricted to genes showing statistically significant expression differences with a fold change ≥2 between PR1 and PR2/PR3 in pioneer root samples and between PS1 and PS2/PS3 in stem samples.

### Multiple sequence alignment and phylogenetic analysis

Multiple sequence alignment of AtJAO-related proteins from dicot and monocot model plant species was performed using the Muscle algorithm in MEGA 7.0 (Kumar et al. 2016). A phylogenetic tree was constructed using the neighbor-joining method with 500 bootstrap replicates in MEGA 7.0.

### MeJA-induced expression of oxygenases

MeJA treatment of *Populus* plants was performed as described by Caarls et al. (2017), with modifications. Two-month-old *P. trichocarpa* seedlings were immersed for 5 min in a solution containing 0.2 mM MeJA and 0.015% Silwet. Control plants were treated in parallel with a mock solution lacking MeJA. After 3.5 h, leaves, roots, and stems were harvested. RNA isolation and quantitative real-time polymerase chain reaction (qRT-PCR) were performed as described below. Gene expression levels were calculated relative to control plants treated with 0.015% Silwet and harvested at the same time point. Statistical significance was evaluated using a *t*-test, with triple asterisks in the figures indicating *P* < 0.001.

### qRT-PCR analysis of gene expression

Total RNA was extracted using the Ribospin Plant Kit (GeneAll Biotechnology Co., Seoul, Republic of Korea) according to the manufacturer’s protocol. For reverse transcription, 1 *μ*g of RNA was converted into cDNA using the High Capacity cDNA Reverse Transcription Kit (Applied Biosystems, Thermo Fisher Scientific Inc., Waltham, MA, USA) following the manufacturer’s instructions. qRT-PCR was carried out using SYBR Green Master Mix (Applied Biosystems, Thermo Fisher Scientific Inc.) on a CFX96 Touch Real-Time PCR Detection System (Bio-Rad Laboratories, Hercules, CA, USA). The amplification program included an initial denaturation at 95°C for 10 min, followed by 40 cycles of a two-step protocol: denaturation at 95°C for 15 s, and annealing/extension at 60°C for 1 min. Gene expression was normalized using three housekeeping genes. The gene-specific primer sequences used for qRT-PCR are listed in Supplementary Table S2. Data were analyzed using the comparative CT method (2^−ΔΔCT^). All the expression measurements were performed in three technical replicates and at least three biological replicates for each experimental variant. Statistical significance was assessed using one-way ANOVA followed by Tukey’s *post hoc* test, conducted with Statistica 14 software (StatSoft Poland Inc., Kraków, Poland).

### Fluorescence *in situ* hybridization

Pioneer roots and stems of *P. trichocarpa* were cut into 35-*μ*m-thick sections using a Leica VT 1200S vibratome (Leica Biosystems, Nussloch, Germany). Sections were fixed in 4% formaldehyde (pH 6.8; Polysciences, Warrington, PA, USA) and 0.5% glutaraldehyde (pH 6.8; Polysciences) for 2 h, rinsed in 0.01 M PBS, and permeabilized with 0.1% Triton X-100 for 15 min. To enhance signal sensitivity, the FISH protocol was combined with tyramide signal amplification (TSA), beginning with a 1-h incubation in 3% hydrogen peroxide to quench endogenous peroxidase activity. Digoxigenin (DIG)-labeled probes (Metabion International AG, Planegg, Germany) were resuspended in hybridization buffer (30% formamide, 4× saline-sodium citrate (SSC), 5× Denhardt’s solution, 1 mM EDTA, and 50 mM phosphate buffer) to a final concentration of 50 pmol. Hybridization was performed at room temperature (RT) for 6 h. The probe sequences are provided in Supplementary Table S2. After hybridization, slices were washed with SSC buffer, blocked in 0.1% acetylated BSA (acBSA) for 30 minutes, and incubated overnight at 8 °C in a humidified chamber with a rabbit anti-DIG primary antibody (1:100; Roche Ltd., Basel, Switzerland) diluted in 0.05% acBSA/PBS. Following PBS washes, slices were incubated for 1 hour at 35 °C with a poly-HRP-conjugated secondary antibody (Thermo Fisher Scientific Inc.) included in the TSA SuperBoost kit. After further PBS washes, the slices were incubated for 15 minutes at RT with a tyramide–Alexa Fluor 488 conjugate, prepared according to the SuperBoost kit protocol (Thermo Fisher Scientific Inc.). Sections were mounted in ProLong Gold (Thermo Fisher Scientific Inc.) and imaged using a Leica TCS SP5 confocal microscope (Leica Biosystems). Autofluorescence of lignin was observed using a 405 nm diode laser, and FISH signals were visualized using 488 nm excitation from an argon laser. Control reactions lacking either the probe or the primary antibody were included to assess specificity (Supplementary Figure S6).

### Immunolocalization of JA

Fragments of stems were fixed in 3% (v/v) *N*-(3-dimethylaminopropyl)-*N*′-ethylcarbodiimide hydrochloride (EDAC) for 2 h, followed by fixation in a mixture of 2% glutaraldehyde (pH 6.8; Polysciences, Warrington, USA) and 2% (v/v) formaldehyde (pH 6.8; Polysciences, Warrington, USA) for 12 h at 4°C. The fixative was then discarded, and the samples were rinsed three times in 0.1 M buffer. Sections (30 *μ*m) were prepared using a vibratome Leica VT 1200S. JA was localized using primary anti-JA rabbit antibodies (Agrisera, Sweden, catalog number AS11 1799) at a dilution of 1:500. Immunolocalization assays were performed as described by Wojciechowska et al. (2018). Results were recorded using a Leica Stellaris DMi8 confocal microscope (Leica Biosystems) utilizing a white light laser (WLL) emitting light at 488 nm. In the control reactions, incubations with the primary antibodies were omitted (Supplementary Figure 4g–i).

### Protein structure modeling and preparation

To construct structural models of selected *P. trichocarpa* proteins as potential JA oxidases, the standalone version of AF2 (Jumper et al. 2021) was used. Models were generated using the monomer prediction mode, and the top-ranked structure for each protein was selected for further analysis. The resulting AlphaFold models were prepared for molecular docking using the Protein Preparation Wizard (Sastry et al. 2013). Structure minimization was performed using the Prime module (Jumper et al. 2021) with the OPLS4 force field and default convergence criteria (RMS gradient value of 0.3).

### Preparation of ligands for molecular docking

JA was used as the ligand molecule for docking studies. Ligand preparation was performed using the LigPrep protocol within the Schrödinger Suite (Schrödinger, LLC, New York, NY, USA). Tautomeric and ionization states were generated using the Epik module (Greenwood et al. 2010).

### Binding site and grid generation

The grid box for docking was positioned over the binding pocket of the JAO2/JOX2/JA complex in the crystal structure with PDB code 6LSV (Zhang et al. 2021). Grid generation was carried out using the Glide module (Friesner et al. 2006). The prepared grid box retained Fe^3+^ and 2-OG within the binding site for docking simulations.

### Molecular docking

IFD was used for docking simulations (Sherman et al. 2006), combining Glide with the refinement module in Prime (Jacobson et al. 2002). This protocol accounts for conformational changes in the protein upon ligand binding. Initial docking of each ligand was performed using a softened potential with van der Waals radii scaling set to 0.5 for both the receptor and ligand. Residue side chains within 7 Å of any ligand pose were refined, and subsequent minimization was applied to each protein–ligand complex. Final docking of the ligand into the refined receptor structure was performed using Glide in SP mode (Halgren et al. 2004). Grid boxes for the IFD protocol were generated based on JA orientation in the 6LSV structure. The default IFD protocol was used, with a maximum of 20 poses generated per ligand. Each resulting ligand pose was rescored using Molecular Mechanics – Generalized Born Surface Area (MM-GBSA) binding energy estimation with the Prime module. The binding energy was calculated as the difference between the energy of the protein–ligand complex and the sum of the energies of the unbound protein and ligand. Entropy contributions were not included. The variable-dielectric generalized Born (VSGB) solvation model (Li et al. 2011) was used with the OPLS4 (optimized potentials for liquid simulations) force field (Lu et al. 2021), and a minimized sampling method was applied. Each docking pose of the JA ligand was compared by calculating the RMSD value relative to the experimental ligand pose in the 6LSV PDB entry.

### Molecular dynamics

Molecular dynamics simulations were performed using the Desmond package (Desmond Molecular Dynamics System, 2023). Each system was simulated at 300 K and 1 bar pressure using an NPT (constant-temperature, constant-pressure) ensemble with a Nose–Hoover thermostat and Martyna–Tobias–Klein barostat. The integration time step was 2 fs, and the SHAKE algorithm was used to constrain hydrogen–heavy atom bond lengths. Long-range electrostatic interactions were handled with the smooth particle mesh Ewald method, and a 9 Å cutoff was applied to short-range Coulomb interactions. Before simulation, each system was solvated in an orthorhombic box using the transferable intermolecular potential with 3 points (TIP3P) water model, neutralized with counterions, and supplemented with 0.15 M NaCl. The OPLS4 force field was used throughout all the simulations. Data were analyzed using tools implemented in Maestro (Schrödinger Suite), focusing on ligand–protein interactions and complex stability, with particular attention to RMSD and RMSF (root mean square fluctuation) profiles.

### Protein overexpression and purification

pRSFDuet-1 expression vectors encoding Potri.006G101200 or Potri.009G022800 with an N-terminal 6× His tag were obtained from commercial vendors (Biomatik Corporation, Wilmington, DE, USA) and transformed into *E. coli* BL21(DE3)pLysS. Cultures were grown at 37°C to an OD_600_ of 0.6–0.8 (~2 h) and induced with 0.5 mM isopropyl-β-d-1-thiogalactopyranoside (IPTG) for an additional 4 h at 37°C (Janicki et al. 2020). Cells were harvested by centrifugation, and the pellets were resuspended in lysis buffer supplemented with a protease inhibitor cocktail (Roche) and sonicated on ice. After a second centrifugation, the supernatants were loaded onto Ni-NTA (Nickel-nitrilotriacetic acid) columns (Invitrogen, Thermo Fisher Scientific Inc.) pre-equilibrated according to the manufacturer’s protocol. Columns were washed three times with buffer containing 50 mM Tris–HCl (pH 7.5) with increasing NaCl (100–200 mM) and imidazole (20–40 mM) concentrations. Recombinant proteins were eluted with 300 mM imidazole, and the eluted fractions were pooled and concentrated using Amicon centrifugal filters (Merck KGaA, Darmstadt, Germany) to a final volume of ~0.5 mL. Purity was assessed by sodium dodecyl sulfate–polyacrylamide gel electrophoresis (SDS–PAGE) followed by Coomassie Brilliant Blue staining, and protein concentrations were determined using the Bradford assay. The same purification workflow was applied to both Potri.006G101200 and Potri.009G022800.

### Enzyme assay

Enzyme activity assays were conducted under conditions adapted from previously established protocols for 2-OG-dependent oxygenases (Smirnova et al. 2017, Ndecky et al. 2025). Each reaction (200 *μ*L) contained 20 *μ*g of purified recombinant *Populus* oxygenase protein, 50 *μ*M JA, 50 mM Tris–HCl pH 7.0, 5 mM dithiotreitol, 13.3 mM 2-OG, 13.3 mM ascorbate, 0.67 mM FeSO₄, and 200 *μ*g bovine serum albumin (BSA). The enzymatic reaction was incubated at 28°C for 1 h. Reactions were terminated by adding 1/5 volume of 1 M HCl, followed by extraction with one volume of ethyl acetate. The organic phase was collected, evaporated under a nitrogen stream, and resuspended in 150 *μ*L of 50% methanol. Reaction products were subsequently analyzed using liquid chromatography–tandem mass spectrometry (LC–MS/MS) as described by Smirnova et al. (2017) to detect JA and its hydroxylated derivative (OH-JA).

### Anatomical studies

Fragments of stems and roots were fixed in a solution containing 2% glutaraldehyde (pH 6.8; Polysciences) and 2% formaldehyde (pH 6.8; Polysciences), and embedded in Technovit 7100 resin (Kulzer GmbH, Hanau, Germany) following the protocol described by Wojciechowska et al. (2018). Cross-sections were prepared using a Leica RM2265 Fully Automated Rotary Microtome (Leica-Reichert, Wetzlar, Germany) at a thickness of 10 *μ*m. Sections were stained with 0.5% toluidine blue and examined under a light microscope (Axioscope A1, Carl Zeiss Microscopy GmbH, Oberkochen, Germany). Anatomical measurements were performed using Zeiss Zen 3.2 software (Carl Zeiss Microscopy GmbH). For each genotype, at least 30 plants were evaluated. Significance levels were determined using a *t*-test and are indicated in the figures: a single asterisk represents *P* < 0.05, and a triple asterisk represents *P* < 0.001.

## Supplementary Material

Supplementary_Figures_pcag035

Supplementary_Tables_pcag035

## Data Availability

The datasets generated for this study can be found in the Gene Expression Omnibus database: accession number GSE126842. The datasets generated and analyzed during the current study are available in the RepOD repository https://repod.icm.edu.pl/dataset.xhtml?persistentId=doi:10.18150/X75FTU.
