# Supplementary material for: Functional insights into a putative 2-OG oxygenase involved in jasmonic acid metabolism and xylem development in Populus trichocarpa
Source: Plant Cell Physiol. 2026 Mar 16;67(8):1299–315. doi: 10.1093/pcp/pcag035 (PMC13545655; doi:10.1093/pcp/pcag035)
Supplement: Supplementary file 1 [file supplementary_figures_pcag035.docx]

Supplementary figure S1. Anatomical analysis of stem and root fragments selected for developmental stage-specific analyses. a-f – *Populus trichocarpa* (a - PS1 - apical meristem with stem primary development; b - PS2 - early secondary growth of stem; c – PS3 - isolated secondary xylem; PR1 - d - root tips with apical meristem; PR2 - e - primary root development; PR3 - f - secondary root development; g-l – *Arabidopsis thaliana* (g – S1 - apical meristem with primary development; h - S2 - early secondary growth of stem; i – S3 - advanced secondary growth of stem; j – R1 - root tips with apical meristem; k – R2 – root primary development; l – R3 – root secondary development). Bars – 200 µm.

Supplementary figure S2 - Structural comparison and sequence alignment of selected *Populus* oxygenases with *Arabidopsis* JAO/JOX proteins. a - Structural superposition of *Potri.006G101200*, *Potri.009G022800*, and *Arabidopsis* JAO/JOX1–4. Structural models were obtained using AlphaFold2; JA orientation is shown as spheres. b - Sequence alignment of *Potri.006G101200*, *Potri.009G022800*, and *Arabidopsis* JAO/JOX1–4. Conserved arginine and phenylalanine residues (the arginine/phenylalanine triad) are marked with red and blue asterisks, respectively.

Supplementary figure S3. Phylogenetic tree constructed in MEGA 7.0 with 500 bootstrap replicates, based on protein sequences of two *Populus* oxygenases, four *Arabidopsis thaliana* (At) JAO/JOX proteins, four *Nicotiana attenuata* (Na) JAO/JOX proteins and four *Oryza stativa* (Os) JAO/JOX proteins. Bootstrap values after 500 iterations are shown on nodes. Protein sequence alignement used for constructing of the tree is shown in Supplementary 5

Supplementary figure S4. Protein sequence alignement used for constructing of the phylogenetic tree

Supplementary figure S5. Immunolocalization of JA (green fluorescence) and lignin distribution (red autofluorescence) in *Populus* stems. a-c – Localization of JA during primary stem growth. d-f – Localization of JA during secondary stem growth. g-i - Negative control reactions without the primary anti-JA antibody. PX - primary xylem, SX - secondary xylem, Ph - phloem, Pf - phloem fibers, PC – cortex parenchyma cells. Scale bars = 100 µm.

Supplementary figure S6. Representative images of negative control reactions omitting either the probe or anti-DIG antibodies. LM – light microscopy image of the same section. Scale bars = 200 µm.
