# Supplementary material for: Functional insights into a putative 2-OG oxygenase involved in jasmonic acid metabolism and xylem development in Populus trichocarpa
Source: Plant Cell Physiol. 2026 Mar 16;67(8):1299–315. doi: 10.1093/pcp/pcag035 (PMC13545655; doi:10.1093/pcp/pcag035)
Supplement: Supplementary file 2 [file supplementary_tables_pcag035.docx]

Supplementary Table S1 – List of potential orthologs of *Potri.006G101200* and *Potri.009G022800* identified in the *Arabidopsis* genome using the Phytozome database.

|  | *Potri.006G101200* | *Potri.009G022800* |
| --- | --- | --- |
|  | **SIMILARITY** | |
| ***AT3G11180*** | 80,9% | 55,4% |
| ***AT5G05600*** | 79,7% | 55,1% |
| ***AT3G55970*** | 77% | 55,1% |
| ***AT2G38240*** | 77% | 58,5% |

Supplementary Table S2. List of primers and probes used in the research.

| **Primers used for qRT-PCR** | | | |
| --- | --- | --- | --- |
| **Species** | **ID** | **Sequence (5'-> 3')** | |
| *Populus trichocarpa* | Potri.006G101200_F | GGGGATCAAATCCAGGTACTCAG | |
|  | Potri.006G101200_R | CTGGTCCTTGGTGAGCAGTT | |
|  | Potri.009G022800_F | TGGGGCAGATCACAGAGATATT | |
|  | Potri.009G022800_R | TTCCACGGTGACAGTCTTGT | |
|  | UBQ_F | AGGAACGCGTTGAGGAGAAG | |
|  | UBQ_R | TATAAGCAAAAACCGCCCCTG | |
|  | ACT_F | GCCCAGAAGTCCTCTTCCAG | |
|  | ACT_R | AAGGGCGGTGATCTCCTTG | |
|  | 18S rRNA_F | AATTGTTGGTCTTCAACGAGGAA | |
|  | 18S rRNA_R | AAAGGGCAGGGACGTAGTCAA | |
|  | TUB_F | TTCTCCTGAACATGGCAGTG | |
|  | TUB_R | CCACACAACGTGAAATCCAG | |
|  | GAPDH_F | CAATGAATGGGGCTACAGGT | |
|  | GAPDH_R | CATGAATCAGCTGCACATCC | |
| *Arabidopsis thaliana* | At5g05600 F | CTCATCCCCATGCTTTCATC | |
|  | At5g05600 R | TCCGAGTTCACTATCACTCTATGC | |
|  | At3g11180 F | CGTGGATCACTGTCAATCCT | |
|  | At3g11180 R | CGATGTTCCACGCTCTTGTA | |
|  | At5g55970 F | GAACCAGCTCCTCATGCTTT | |
|  | At5g55970 R | GGGTTCACGATCACTCTGTG | |
|  | At2g38240 F | ATGGCTGGGTCACCATTAAA | |
|  | At2g38240 R | GTTCCACGCTTTTGTAAATTCC | |
|  | UBQ_F | GTGGTGCTAAGAAGAGGAAGA | |
|  | UBQ_R | TCAAGCTTCAACTCCTTCTT | |
|  | ACT F | GGTAACATTGTGCTCAGTGG | |
|  | ACT R | CTCGGCCTTGGAGATCCACA | |
|  | GAPDH F | TTGGTGACAACAGGTCAAGCA | |
|  | GAPDH R | AAACTTGTCGCTCAATGCAATC | |
| **Primers used for genotyping** | | | |
| *Arabidopsis thaliana*  *(joxQ mutant)* | At3g11180 (SAIL_131_D01) F | | GCCTCAACGATCAAGAACAAG |
|  | At3g11180 (SAIL_131_D01) R | | GTTGAGGGCACTTTGGGTAA |
|  | At5g05600 (GK_870C04) F | | CTCATCCCCATGCTTTCATC |
|  | At5g05600 (GK_870C04) R | | CGAGGAGAAATATGAGATTCAACA |
|  | At3g55970 (SAIL_861_E01) F | | GAACCAGCTCCTCATGCTTT |
|  | At3g55970 (SAIL_861_E01) R | | GGGTTCACGATCACTCTGTG |
|  | At2g38240 (SAIL_268_B05) F | | ATGGCTGGGTCACCATTAAA |
|  | At2g38240 (SAIL_268_B05) R | | CAAGGACCCTTTTGCCTTATC |
|  | SAIL LB (for SAIL_268_B05) | | TAGCATCTGAATTTCATAACCAATCTCGATACAC |
|  | SAIL LB (for SAIL_131_D01 and SAIL_861_E01) | | TCTGAATTTCATAACCAATCTCG |
|  | GABIKAT LB 08409 | | ATATTGACCATCATACTCATTGC |
| **Probes used for FISH** | | | |
| *Populus trichocarpa* | Potri.009G022800 | | DIG- CTTATTATCTCTGTGATCTGCCCCA |
|  | Potri.006G101200 | | DIG-GCTGAGTACCTGGATTTGATCCCC |
